# Water-Induced Tuning of the Emission of Polyaniline LEDs within the NIR to Vis Range

**DOI:** 10.1021/acsomega.1c05051

**Published:** 2021-12-10

**Authors:** Jerzy J. Langer, Katarzyna Ratajczak, Ewelina Frąckowiak, Sebastian Golczak

**Affiliations:** Faculty of Chemistry, Laboratory for Materials Physicochemistry and Nanotechnology, A. Mickiewicz University in Poznań, Uniwersytetu Poznańskiego 6, 61-614 Poznań, Poland

## Abstract

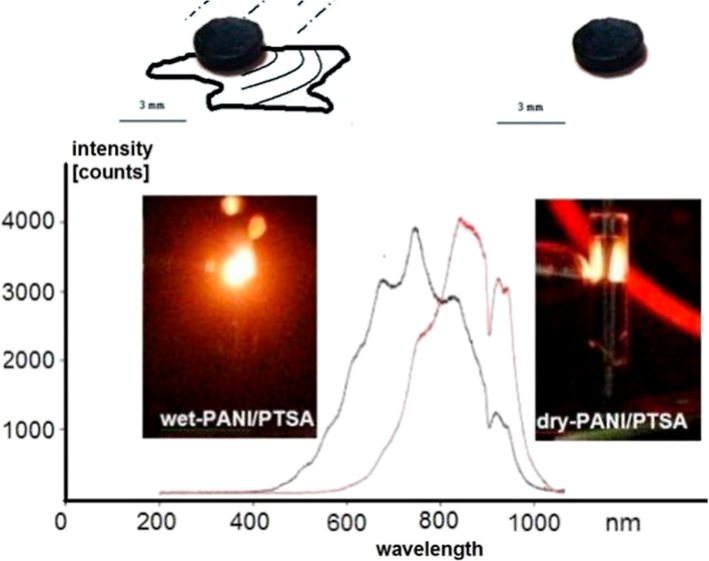

Tuning of the emission within the near-infrared to visible range is observed in *p*-toluenesulfonic acid-doped polyaniline light emitting diodes (PANI/PTSA), when water molecules are absorbed by the active material (wet PANI/PTSA). This is a hybrid material that combines a conjugated π-electron system and a proton system, both strongly interacting in close contact with each other. The proton system successfully competes with the electron system in excitation energy consumption (when electrically powered), thanks to the inductive resonance energy transfer from electrons to protons in wet PANI/PTSA at the energy levels of combination of vibrations and overtones in water, with subsequent light emission. Wet PANI/PTSA, in which electrons and protons can be excited parallelly owing to fast energy transfer, may emit light in different ranges (on a competitive basis). This results in intense light emission with a maximum at 750 nm (and the spectrum very similar to that of an excited protonic system in water), which is blue-shifted compared to the initial one at ∼850 nm that is generated by the PANI/PTSA dry sample, when electrically powered.

## Introduction

Electron transfer, proton transfer, and excitation energy transfer from electrons to protons with excitation of the proton system are of great importance for key biological processes and modern advanced technological applications^1−19^ as both intramolecular processes^2,3,6,9,12,17,21^ and external, intermolecular, and intersystem ones.^3,8,11−14,17,19,21^ The last case is particularly interesting in connection to technical applications,^11,14,18^ but they also have a crucial role in functioning biological systems at cellular and sub cellular levels.^1,3,7,8,13,14,18^ Such complex problems and systems are very often successfully examined with experimental and theoretical models, supported by computer simulations.^5−7,9,17,19^

A unique model material for studying some aspects of these processes by electroluminescence^20,21^ is *p*-toluenesulfonic acid-doped polyaniline (PANI/PTSA). This is due to the presence of a conjugated π-electron system with relatively high electrical conductivity and an interacting with it coupled hydrogen bonding system, which can be modified by the presence of water molecules (Figure 3a).

The emission of light by conductive polymers that are electrically powered has been of our interest for over 10 years using macroscopic samples (∼1 mm) in experiments, instead of thin layers with a thickness of ∼1 μm, unlike in other laboratories.^20^ We described polyaniline light-emitting diodes (LEDs) with non-linear effects, including stimulated Raman scattering^21^ and polyaniline lasing,^22^ and also the electroluminescence of polypyrrole.^23^

On the other hand, we have discovered emission of light in the entire range of ultraviolet–visible–near-infrared (UV–vis–NIR) due to the excitation of protons in the protonic analogue of the p–n junction—protonic LED,^24^ formed in water as a protonic semiconductor, appropriately doped.^25,26^

In this paper, we describe the unique light emission observed in polyaniline doped with *p*-toluenesulfonic acid (PANI/PTSA), which is modified (tuned) in the range from 850 nm (NIR) to 750 nm (vis) in the presence of water.

The emitter is a hybrid material that combines conjugated π-electrons and a coupled proton system, both strongly interact with each other, while remaining in close contact, so that electrons and protons can be simultaneously excited due to energy transfer, when the system is electrically powered.

## Results and Discussion

This work is devoted to unique properties of polyaniline doped with *p*-toluenesulfonic acid, PANI/PTSA, a hybrid model material, where electrons and protons are excited when electrically powered, emitting the light in different ranges in a competitive way. The curiosity is that the protonic system starts to be active (effective in emission) in the presence of water and it is competitive despite emitting the light of higher photon energy (blue-shifted). The final result resembles a photon upconversion—NIR emission transits into the vis range—but the mechanism is different.

**Figure 1:**
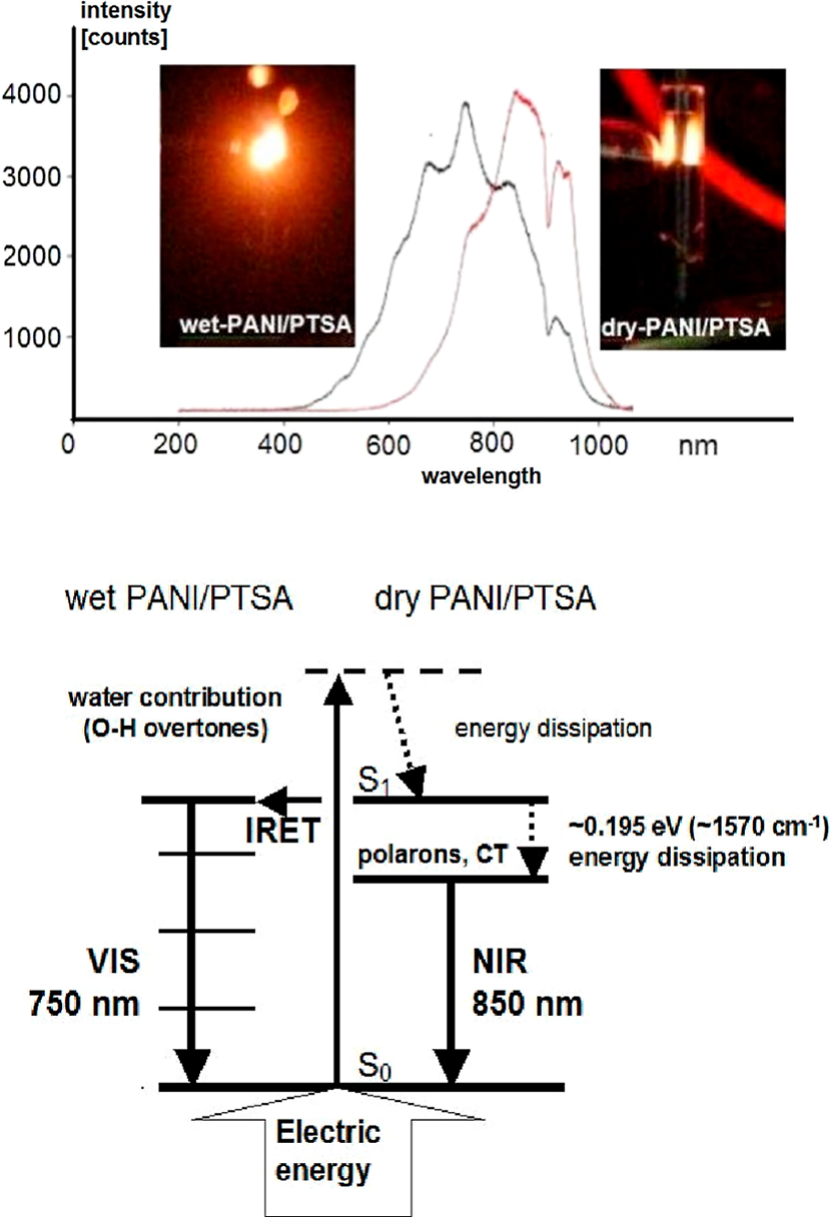
Comparison of PANI/PTSA electroluminescence spectra in the dry state (right spectrum) and in the presence of water (left spectrum), and a diagram of the processes involved.

**Figure 2:**
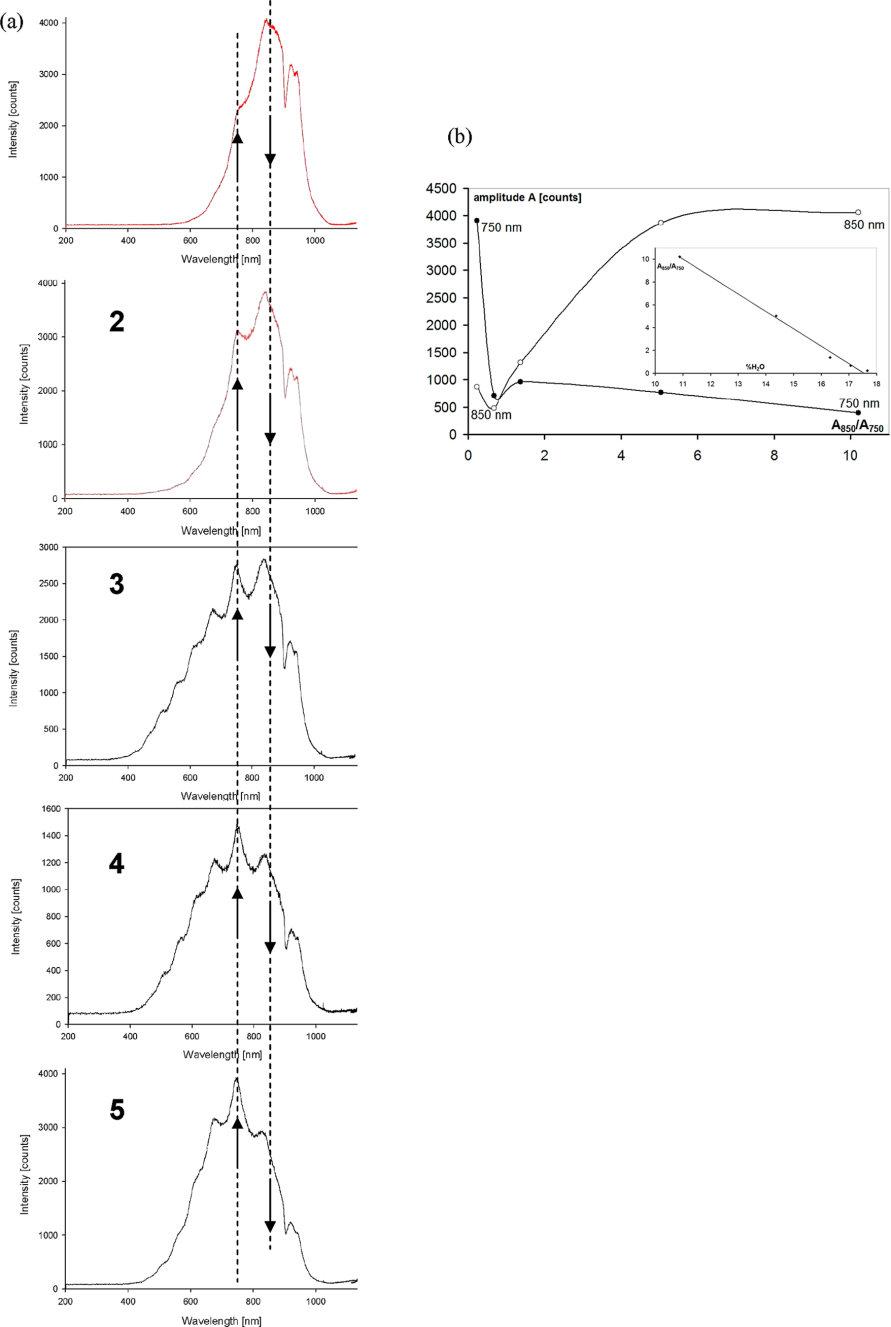
Evolution of PANI/PTSA electroluminescence spectra with increasing water content: sample **1**, 10.9% H_2_O; sample **2**, 14.4% H_2_O; sample **3**, 15.9% H_2_O; sample **4**, 17.1% H_2_O; and sample **5**, 17.7% H_2_O (a). Changes in the spectral amplitudes of the emission at 750 and 850 nm (the emission at 750 nm is typical for wet PANI/PTSA and that at 850 nm corresponds to dry PANI/PTSA) as a function of the amplitude ratio of *A*_850nm_/*A*_750nm_; the ratio *A*_850nm_/*A*_750nm_ linearly correlates with the water content—inset (b).

The diode formed with dry polyaniline doped with *p*-toluenesulfonic acid (solid pressed pellet, with a thickness of 0.5 ± 0.01 mm and a diameter of 3 mm) emits mostly in NIR with a maximum at 840–885 nm (Figures 1 and 2a). This corresponds to the excitations of π-electrons and emission due to the charge-transfer (CT) processes in organic materials.^20,27−29^ Here, CT between PTSA and polyaniline and also between quinoid and aromatic moieties in polyaniline chains is to be considered, including the formation of polarons that are weakly emissive.^30^

**Figure 3:**
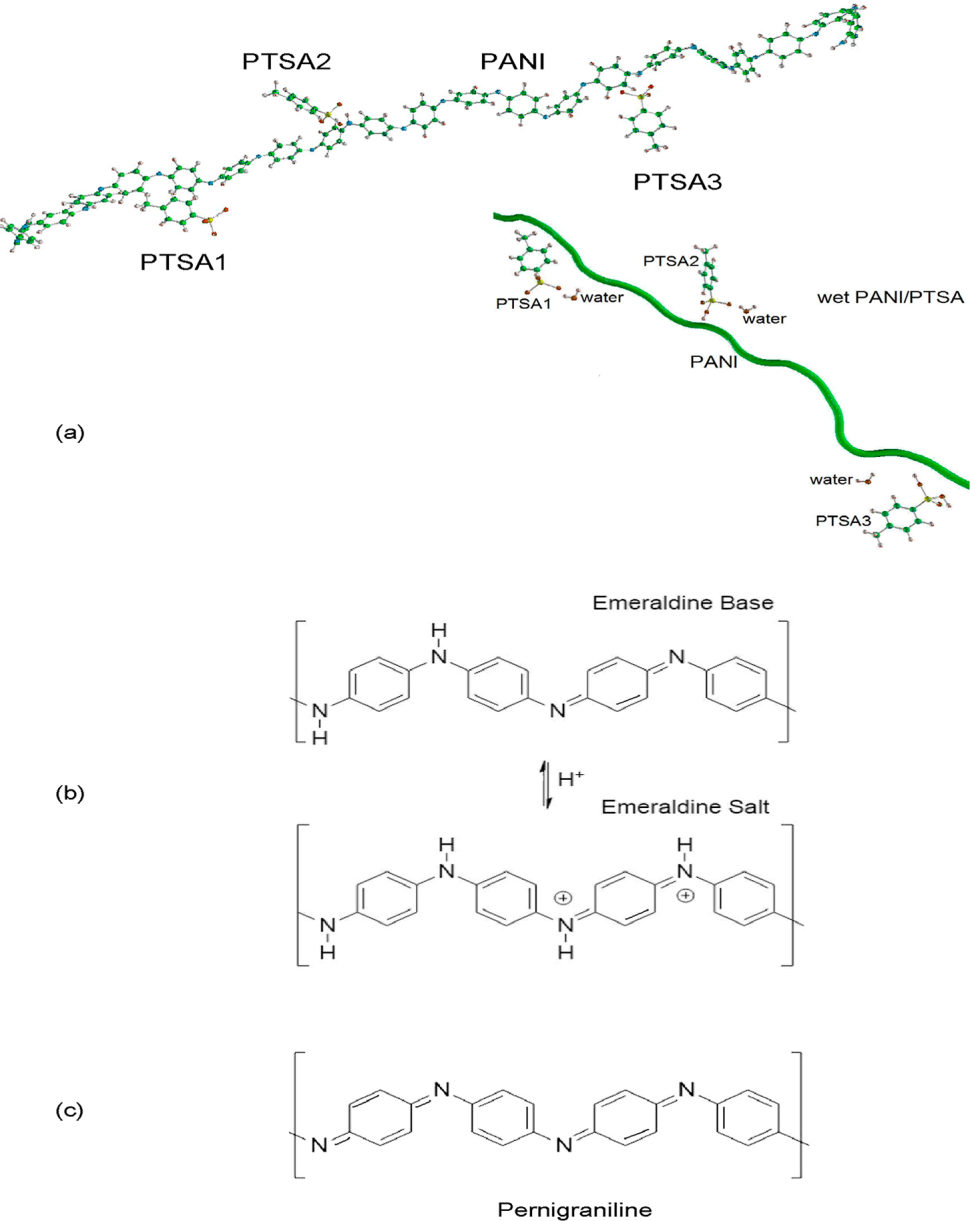
Model of the PANI/PTSA complex; inset: wet PANI/PTSA (a), emeraldine base and emeraldine salt (b), and pernigraniline (c).

PTSA can interact with PANI in two ways:

- due to Coulomb forces of negative and positive charges of anionic −SO_3_^–^ and cationic −N–H^+^ groups, respectively (Figure 3a, TPSA2 and TPSA3);
- due to non-polar forces, originating from interactions of π-electron aromatic rings (Figure 3a, TPSA1), which are particularly adequate for the CT process; in addition, these are responsible for lowering the energy of the electron excited state and the energy of photons generated, leading to emission in the NIR region with a maximum at 845–885 nm; electroluminescence of PANI/HCl, polyaniline doped with HCl—with no π-electron interactions, is observed as broad bands at 460, 575, and 657 nm, with a maximum at 575 nm.^21^

### Influence of Water

With the increase in the water content (humidity of the active material—polyaniline doped with *p*-toluenesulfonic acid), a shift of the maximum emission toward the blue and an increase in the light intensity in a part of the spectrum around 750 nm are observed, while the emission intensity at 845–885 nm decreases clearly (Figures 1 and 2a,b).

Generally, the high dielectric constant of water should result in a bathochromic shift of the emission,^31^ which is not observed. On the other hand, the observed blue shift of 100 nm is too large to be considered a typical hypsochromic effect related to the protonation of nitrogen atom n-electrons due to polyaniline hydration. In addition, there is no pure n−π transition identified in polyaniline, and the spectral range considered corresponds to CT and polaron bands.^31,32^ This indicates another mechanism—an effective transfer of the excitation energy (originally provided by the electric current) from the electron system of polyaniline into the protonic one.^3^ The energy transfer to the coupled protonic system^37−39^ is fast and effective enough so that the light emission from an excited protonic system^24^ takes place as a competitive process, which is similar in mechanism to the generation of polaritons owing to strong coupling, when the resonant energy exchange between a confined optical mode and a material transition is faster than any decay process.^44^ In consequence, the loss of energy is lower than in the case of the excited polyaniline electronic states. This results in a blue-shifted spectrum and more intensive light emission. In wet PANI/PTSA, owing to inductive resonance energy transfer (IRET) from electrons to protons, the provided electrical energy excites the protonic system up to the energy levels of combination vibrations and overtones in water-coupled hydrogen bonding^3,24^ with subsequent emission of light at 750 nm. This is a unique behavior. Usually, the hydrogen bonding system is responsible for the dissipation of the excitation energy due to the rapid energy transfer between the electron and proton systems, followed by the energy flow in the hydrogen bonding network, and consequently the relaxation of electronic excitations through conjugated hydrogen bonds.^3,33−37^

### Dissipation and Transfer of the Excitation Energy

The light emission from the protonic system excited owing to IRET from electrons to protons is a new phenomenon, which dominates in experiments performed with wet PANI/PTSA (Figure 2a,b). The emission spectrum consists of two components: the contribution of the excited basic electron system of dry PANI/PTSA with a maximum at ∼850 nm and the excited protonic system (including water) at ∼750 nm in wet PANI/PTSA, which are additive in a competitive way (Figures 1 and2a,b). In both cases, despite the emission of light, the dissipation of the excitation energy also takes place in non-radiative processes.^3^

This is particularly effective when the electron and proton systems are involved at a comparable level in consuming the excitation energy, that is, for the emission amplitude ratio of *A*_850nm_/*A*_750nm_ equal to 1 (Figure 2b). The proton system is not yet ready and effective enough in emission, but both channels dissipate the energy, leading to the lowest light emission.

Generally, in polyaniline, there is a strong coupling between electrons and protons, including the protons of the absorbed water.^31^ Protonation essentially influences the PANI π-electron system and the electrical conductivity (e.g., emeraldine salt, Figure 3b). This enables efficient energy transfer and excitation the protonic system, when polyaniline electrons are excited owing to the electric current flow.

### Electrical Conductivity

**Figure 4:**
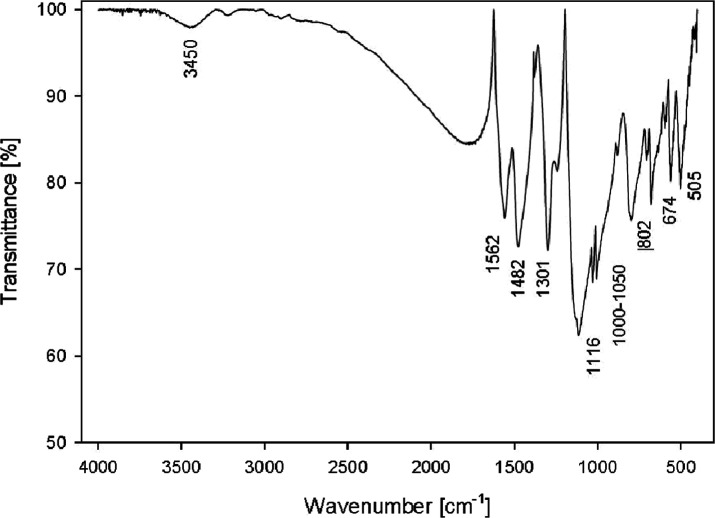
FTIR spectrum of dry PANI/PTSA (B—benzenoid rings; Q—quinoid rings): 3450 cm^–1^, N–H stretching mode; 1562 and 1482 cm^–1^, C=C stretching of Q and B; 1301 cm^–1^, C–N stretching of the secondary aromatic amine; 1116 cm^–1^, a strong band ascribed to the electronic absorption of N=Q=N (40) and C–H in-plane bending vibration (mode of N=Q=N, Q=N + H–B, and B–N + H–B); 802 cm^–1^, out-of-plane deformation of C–H in the 1, 4-substituted benzene ring; 505 cm^–1^, C–H deformation of an aromatic ring; and 1000–1050 cm^–1^ and 674 cm^–1^, −SO_3_ vibrations.

**Figure 5:**
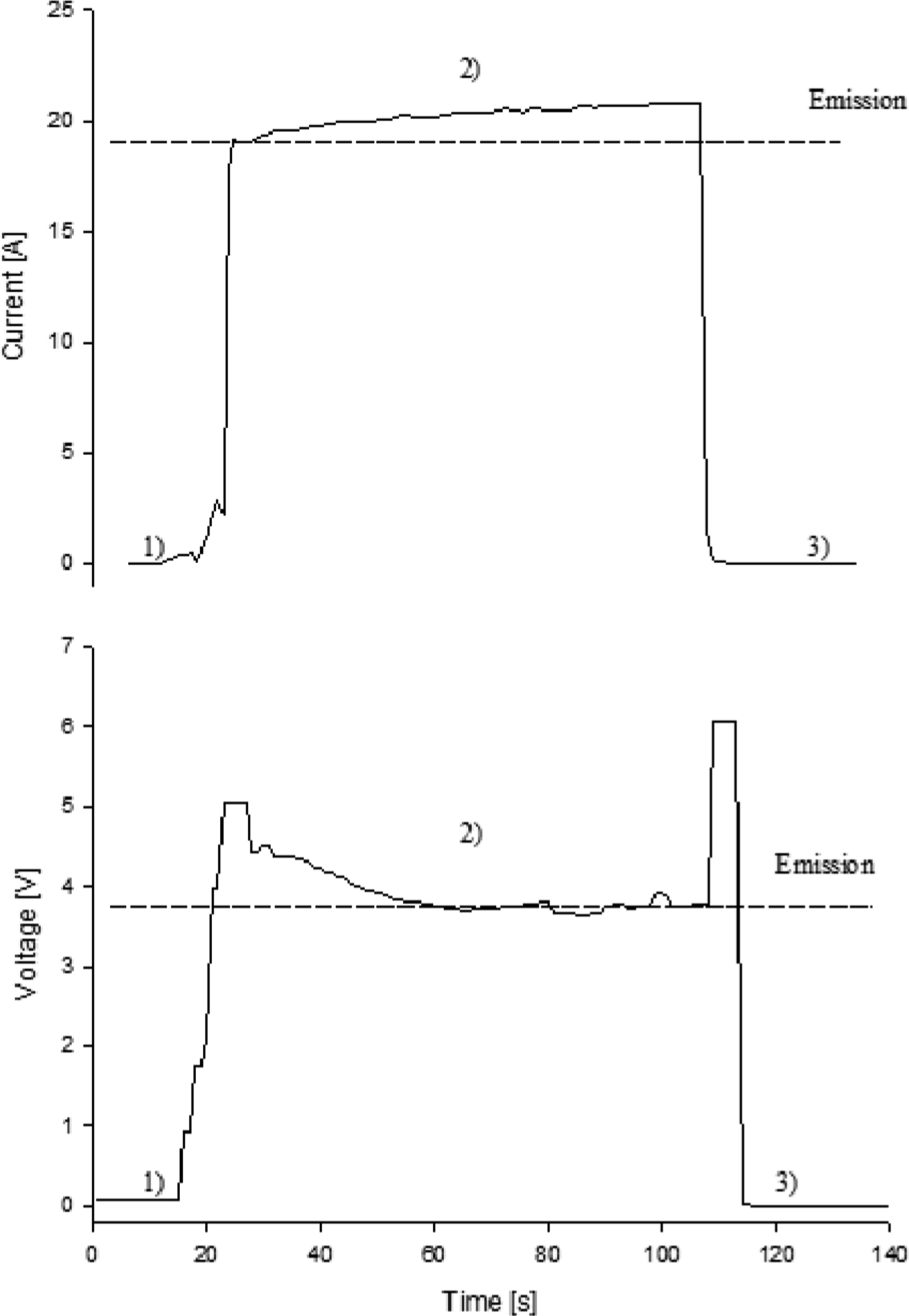
Typical current and voltage changes as a function of time, recorded before (1), during (2), and after the emission (3) for dry PANI/PTSA; the dashed line shows the minimum operating voltage of 3.74 V and the current of 18 A.

A well-defined emission from dry PANI/PTSA with the lowest quantum photon energy of 1.401 eV and a wavelength of 885 nm corresponds to the polaron band.^30^ The polyaniline used is a highly conductive material, mainly in the form of an emerald salt (Figure 3b) with an electrical conductivity of 0.8–3.8 S/cm (measured directly in our experiments) and a low energy gap of 0.3 eV, comparable to the data already published 0.2–0.5 eV.^41^ This corresponds to the Fourier-transform infrared (FTIR) absorption attributed to the electron transition—an intensive wide band centered at 1116 cm^–1^ (Figure 4). The optical energy gap corresponding to the vis–NIR emission spectrum of dry PANI/PTSA is higher and amounts to 1.934 eV. The pernigraniline fraction (Figure 3c) with a lower conductivity and a band gap of about 2 eV is expected to exist as domains that act as emission centers. The domain structure of polyaniline has been described previously.^42,43^ In our experiments, the high electrical conductivity of the polyaniline matrix is necessary to achieve the threshold emission current of 7.7 A at 3.04 V, followed by the operating current of 18 A (minimum) at 3.84 V (Figure 5). Switching ON the emission is rapid and reversible with a voltage change of 0.8 V. The PANI/PTSA electrical conductivity varies from 0.7 to 1.0 S/cm (at “OFF”) to 3.7–3.8 S/cm when emitting light. The process is repeatable and reproducible.

### Stability of the Active Material

**Figure 6:**
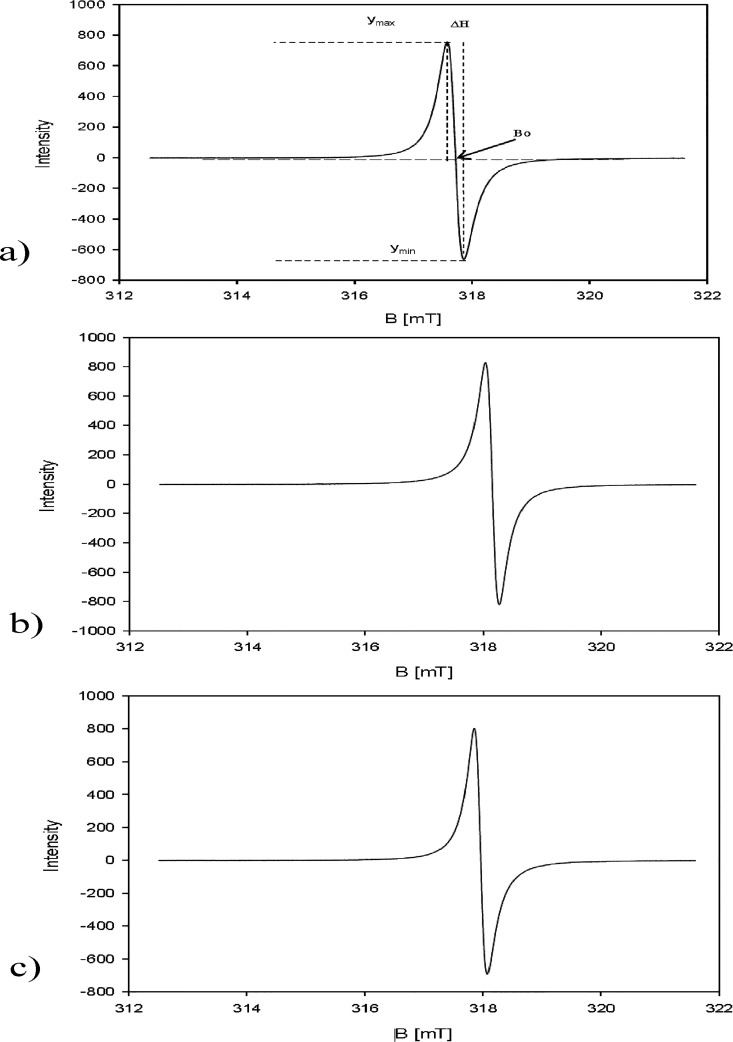
EPR spectra of PANI/PTSA: (a) PANI/PTSA before experiments (powder), *g*-factor = 2.00292, line width Δ*H* [mT] = 0.285, asymmetry *A* = 1.13; (b) crushed dry pellets of PANI/PTSA after light emission, *g*-factor = 2.00299, line width Δ*H* [mT] = 0.236, asymmetry *A* = 1.01; and (c) crushed wet pellets of PANI/PTSA after light emission, *g*-factor = 2.00291, line width Δ*H* [mT] = 0.228, asymmetry *A* = 1.16.

Despite sudden changes in the current (and the electrical conductivity, e.g., 0.76, 3.8, and 1.05 S/cm—before, during, and after emission, respectively), the material stays relatively stable with respect to its electronic structure and electrical properties (Figure 5), including the characteristic parameters measured using the electron paramagnetic resonance (EPR). The EPR signals are very strong for all samples examined (Figure 6), which indicate a high concentration of polarons (dominating charge carriers in polyaniline). Each of the spectra consists of a single narrow Lorentzian line, with the asymmetric factor between 1.01 (dry PANI/PTSA) and 1.16 (wet PANI/PTSA). The values of *g*-factor lie in a narrow range from 2.00291 (wet PANI/PTSA) to 2.00299 (dry PANI/PTSA) and correspond to “free” electrons. The EPR line width after emission is almost the same for dry and wet PANI/PTSA (Δ*H* [mT] = 0.236 and 0.228, respectively) and a bit lower than that for the starting material (Δ*H* [mT] = 0.285). Thus, the electronic structure and interactions did not change essentially.

### Tuning the Emission

**Figure 7:**
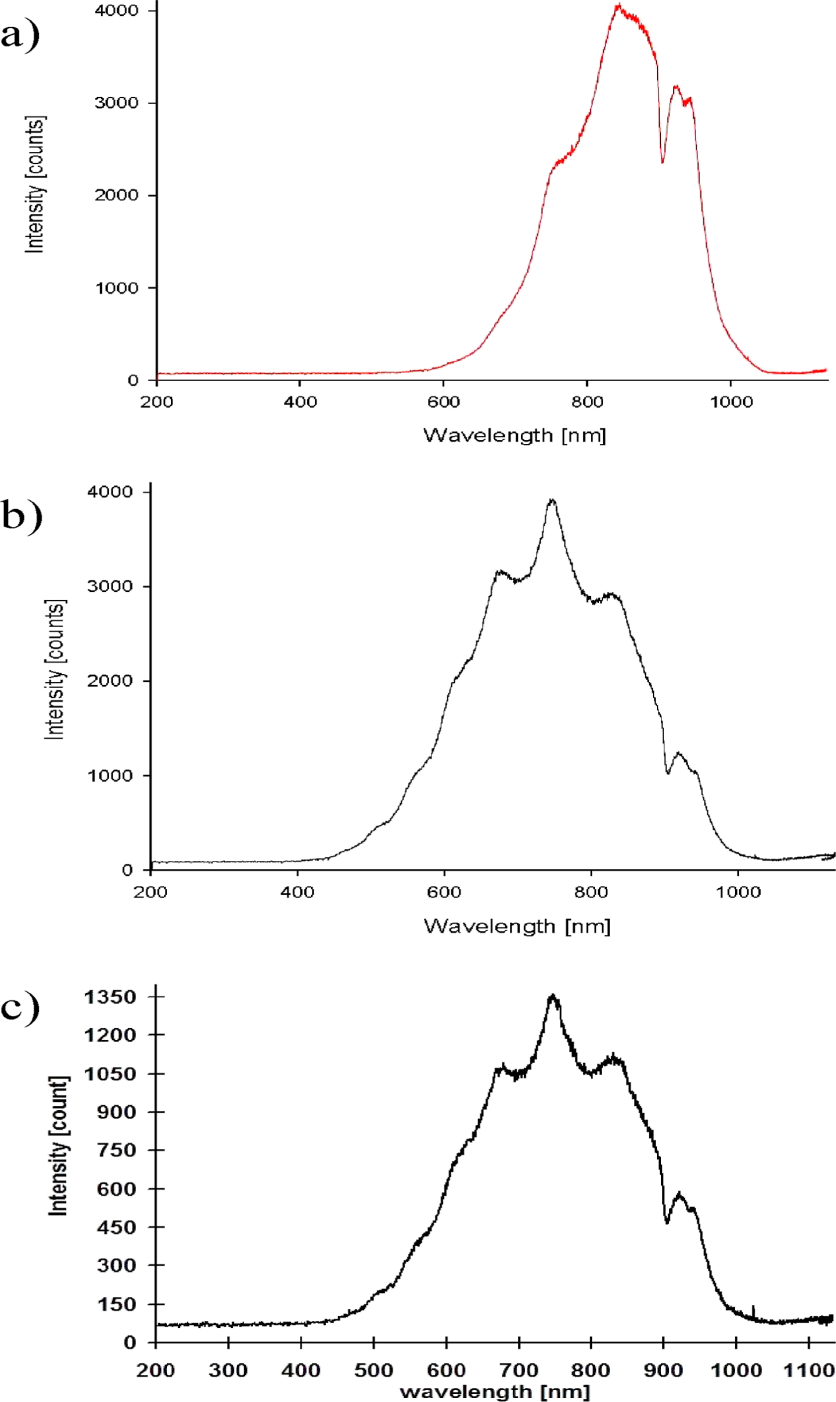
PANI/PTSA electroluminescence spectra in the dry state (a) and in the presence of water—wet PANI/PTSA (b); emission from water doped with sulfonated polystyrene^24^ (c).

Interestingly, the fast energy transfer from the electronic system to the proton system^3^ in the wet PANI/PTSA (Figure 1) limits non-radiative energy dissipation. Polarons and bipolarons, generated in a polyaniline π-electron system (Figure 6) are non-light-emitting quasi-particles,^32^ and energy can be dissipated by molecular vibration. On the other hand, excitation of a protonic system is efficient in light emission, as previously observed.^24^ There is a convincing similarity between the electroluminescence spectra of sulfonated polystyrene doped water^24^ and the protonic system contribution at 750 nm in the current wet PANI/PTSA experiments (Figure 7). Thus, the process of exciting a protonic system and then emitting light is efficient and effectively competitive with the non-radiative energy dissipation from the excited electronic system of polyaniline.

Due to the efficient electron-to-proton energy transfer and the excitation of the protonic system, less dissipation of the supplied energy leads to the emission of photons of greater energy than an excited polyaniline π-electron system, when electrically powered. The difference in the energy of the photons at 750 and 850 nm is ∼0.195 eV (0.19448 eV), which corresponds to the excitation of molecular vibrations in polyaniline, for example, aromatic rings and quinoid grouping at ∼1570 cm^–1^.^38−40^ They have a strong influence on the electron energy (they partially absorb it and dissipate it) but are not involved in the excitation of the proton system, so the energy is not dissipated in this way after a fast transfer (Figure 1), eventually leading to a blue shift in the emission spectrum in the presence of water (wet PANI/PTSA).

Changes in the emission spectrum (estimated roughly by the amplitude ratio at 850 and 750 nm) depend on the sample moisture (Figure 2). In this way, the emission spectrum can be modified by gradually shifting the emission from the NIR to the vis range.

**Figure 8:**
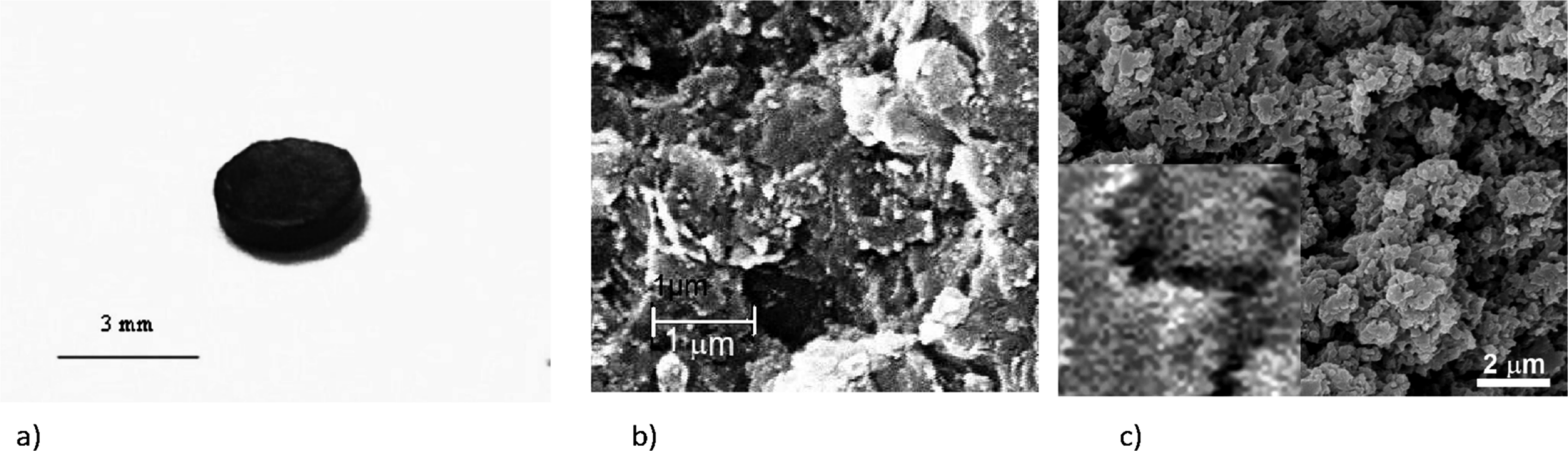
Sample examined (a) and its cross section observed by SEM (b); raw micro- and nanostructured PANI/PTSA, SEM image 5000×; inset: zoomed-in view (c).

This process is expected to be particularly effective when using micro- and nanostructured light-emitting materials, as in our case (Figure 8c), due to the easy diffusion and good contact between water molecules and the active material—here, PANI macromolecules. The effect depends on the ability of the proton system (in water) to interact with the electron system, which is more effective for a micro- and nanostructured material.

## Conclusions

Fast transfer of excitation energy from π-electrons to protons in conjugated hydrogen bonds and effective excitation of the proton system in a hybrid material with strongly coupled electron and proton systems (wet PANI/PTSA) lead to emission of photons with higher energy than the π-electron system in dry PANI/PTSA, when the sample is electrically powered. The water proton system, incorporated into the wet PANI/PTSA, effectively competes with the electron system in terms of excitation energy consumption, resulting in blue-shifted light emission due to lower energy dissipation. As the amount of water absorbed increases, the initial infrared emission at ∼850 nm (NIR) from the dry PANI/PTSA gradually shifts toward the vis range to reach ∼750 nm for the wet PANI/PTSA.

Apart from the possibility of tuning the emission spectrum, the fast and effective transfer of the excitation energy from the electron system to the proton system at the level of the overtones of fundamental oscillations in water molecules (regardless of the source of the excitation energy) is very important for basic biological processes^3^ and also for some modern technical applications, for example, water splitting for fuel production.^3^ This makes our results potentially even wider.

## Methodology

### Materials and Methods

Polyaniline was prepared by oxidation of aniline hydrochloride (10% in water at pH about 1) with the chemical method described elsewhere, modified in our laboratory.

The polymeric material (polyaniline) was characterized with physical and chemical methods: FTIR, EPR, elemental analysis, and electrical conductivity measurements, giving results similar to the values measured for materials previously prepared in our laboratory.^21,22,45^

Chemicals and solvents:

Hydrochloric acid (HCl) (Stanlab, pure p.a.)—600 mL of 1 M solution prepared from concentrated hydrochloric acid (36%)—50 mL of 36% HCl dissolved in 550 mL of H_2_O.

Aniline hydrochloride (C_6_H_8_NCl) (Fisher Scientific, pure p.a.)—6.9 g of aniline hydrochloride dissolved in 300 mL of 1 M hydrochloric acid.

Ammonium persulfate [(NH_4_)_2_S_2_O_8_] (Chempur, pure p.a.)—11.4 g of ammonium persulfate was dissolved in 200 mL of 1 M hydrochloric acid.

Ammonium hydroxide (NH_4_OH) solution 25% (POCh, pure p.a.)—500 mL of 1 M solution prepared from concentrated ammonium hydroxide (25%)—37 mL of 25% ammonium hydroxide dissolved in 463 mL of H_2_O.

Chloroform (CHCl_3_) (Chempur, pure p.a.)—75 mL.

*p*-Toluenesulfonic acid (CH_3_C_6_H_4_SO_3_H) (Aldrich, p.a.)—1.4 g of *p*-toluenesulfonic acid was dissolved in 75 mL of chloroform.

#### Polyaniline Emeraldine Base, PANIEB

Aniline hydrochloride (6.9 g) was dissolved in 300 mL of 1 M hydrochloric acid. At the same time, 11.4 g of ammonium persulfate was dissolved in 200 mL of 1 M hydrochloric acid (separately).

The ammonium persulfate solution was slowly added to a solution of aniline hydrochloride. The resulting dark-green solution was stirred with a magnetic stirrer at room temperature for 24 h.

The precipitate was filtered under reduced pressure and washed several times with distilled water until the filtrate was nearly colorless and neutral. The solid product was treated with 500 mL of 1 M ammonium hydroxide and stirred with a magnetic stirrer at room temperature for 20 h.

The precipitate was isolated by filtration under reduced pressure and washed several times with distilled water until pH ∼ 7. The dark-blue precipitate of the emeraldine base (PANIEB) was dried under ambient conditions (yield 1.8 g).

Elemental analysis:

Found [%]: C 74.05, H 5.01, N 13.61, O 7.33.

Calculated [%]: C 72.69, H 5.09, N 14.13, O 8.07.

[C_12_H_8_N_2_·H_2_0]_*n*_, where one molecule of H_2_O is associated with two monomeric units of C_6_H_4_N.

#### Polyaniline Protonated with *p*-Toluenesulfonic Acid, PANI/PTSA

1.4 g of *p*-toluenesulfonic acid was dissolved in 75 mL of chloroform. Then, 0.9 g of PANIEB was added to this solution in small portions. The mixture was stirred with a magnetic stirrer for 2 h. The dispersion was dripped into a beaker containing 200 mL of distilled water while stirring all the time. The precipitate was filtered under reduced pressure and washed several times with distilled water until pH ∼ 4.5 has been obtained. To remove most water from the product, it was washed several times with methanol. The obtained polyaniline protonated with *p*-toluenesulfonic acid (PANI/PTSA) was dried for 24 h at room temperature.

Elemental analysis:

Found [%]: C 61.605, H 5.145, N 8.47, O 18.528, S 6.252.

Calculated [%]: C 60.450, H 5.280, N 8.46, O 19.330, S 6.460.

([3[C_6_H_4_N·H_2_O]·C_7_H_8_SO_3_]_*n*_), where three molecules of H_2_O are associated with three monomeric units of C_6_H_4_N (1:1) and one unit of the acid: CH_3_C_6_H_4_SO_3_H.

FTIR spectra were measured as a suspension in KBr, pressed disc; for example, Figure 4 (B—benzenoid rings; Q—quinoid rings).

Elemental analyses were performed using a model Vario EL III elemental analyzer (Elementar Analysensysteme GmbH, Germany).

FTIR spectra were recorded using the spectrometer model IFS 66/s (Bruker, USA).

The EPR spectra were recorded using an EPR spectrometer model SE/X 2547 (RADIOPAN, Poland).

The emission UV–vis–NIR spectra were registered on-line with a Spectrometer model 2000 Ocean Optics PC2000 at a resolution of 0.5 nm at the same time when the current and the voltage were measured.

The beam profile was pictured directly with a digital camera: Pentax Kr 12.4 MP + 18–55 mm lens.

The measurements were performed under ambient conditions and in a dark room.

The samples, formed at a pressure up to 6000 kG/cm^2^ as pellets with a thickness of 0.4–0.5 ± 0.01 mm and a diameter of 3 mm (Figure 8a), were placed in a measuring holder inside a glass tube with a wall thickness of 1–2 mm between two solid copper electrodes with a diameter of 4.5 mm (or one of 4.5 mm and the second of 3 mm) and a length of 25 mm.^21−23^

Despite being pressed, the material is microporous (Figure 8b), which allows it to absorb water in the case of preparation of wet PANI/PTSA samples by direct contact with distilled water within 24 h or less.

The voltage and the current were measured with an accuracy of at least of 0.1% using a Brymen digital multimeter, model BM859s (computer controlled), and a Metrahit Energy multimeter (computer controlled) with a precision standard resistor of 0.001 Ω for current measurements, respectively. Stabilized power supplies applied: INCO Z-3020—DC voltage source, adjustable between 0.1 and 30 V under a load current of 0–20 A, and INCO Z-5001—DC voltage source, adjustable between 0.1 and 500 V under a load current of 0–1 A.

To simultaneously register the light beam and the emission spectrum, the optical fiber of the spectrometer was mounted on the same side as the photocamera, in most cases parallel to the optical axis of the camera (another configuration was also used). The distance between the sample and the aperture of the optical fiber was 1–3 cm, and the camera was located at a distance of 15–20 cm.^24^
